# Predictive Modeling of Spinner Dolphin (*Stenella longirostris*) Resting Habitat in the Main Hawaiian Islands

**DOI:** 10.1371/journal.pone.0043167

**Published:** 2012-08-24

**Authors:** Lesley H. Thorne, David W. Johnston, Dean L. Urban, Julian Tyne, Lars Bejder, Robin W. Baird, Suzanne Yin, Susan H. Rickards, Mark H. Deakos, Joseph R. Mobley, Adam A. Pack, Marie Chapla Hill

**Affiliations:** 1 School of Marine and Atmospheric Sciences, Stony Brook University, Stony Brook, New York, United States of America; 2 Duke University Marine Laboratory, Division of Marine Science and Conservation, Nicholas School of the Environment, Duke University, Beaufort, North Carolina, United States of America; 3 Pacific Islands Photo-Identification Network, Honolulu, Hawai'i, United States of America; 4 Cetacean Research Unit, Centre for Fish, Fisheries and Aquatic Ecosystems Research, Murdoch University, Murdoch, Western Australia, Australia; 5 Nicholas School of the Environment, Duke University, Durham, North Carolina, United States of America; 6 Cascadia Research Collective, Olympia, Washington, United States of America; 7 Hawai'i Marine Mammal Consortium, Kamuela, Hawai'i, United States of America; 8 Hawai'i Association for Marine Education and Research, Inc., Lahaina, Hawai'i, United States of America; 9 The Dolphin Institute, Honolulu, Hawai'i, United States of America; 10 Marine Mammal Research Consultants, Honolulu, Hawai'i, United States of America; 11 Psychology and Biology Departments, University of Hawai'i at Hilo, Hilo, Hawai'i, United States of America; 12 Joint Institute for Marine and Atmospheric Research, University of Hawai'i at Mānoa, Honolulu, Hawai'i, United States of America; Texas A&M University-Corpus Christi, United States of America

## Abstract

Predictive habitat models can provide critical information that is necessary in many conservation applications. Using Maximum Entropy modeling, we characterized habitat relationships and generated spatial predictions of spinner dolphin (*Stenella longirostris*) resting habitat in the main Hawaiian Islands. Spinner dolphins in Hawai'i exhibit predictable daily movements, using inshore bays as resting habitat during daylight hours and foraging in offshore waters at night. There are growing concerns regarding the effects of human activities on spinner dolphins resting in coastal areas. However, the environmental factors that define suitable resting habitat remain unclear and must be assessed and quantified in order to properly address interactions between humans and spinner dolphins. We used a series of dolphin sightings from recent surveys in the main Hawaiian Islands and a suite of environmental variables hypothesized as being important to resting habitat to model spinner dolphin resting habitat. The model performed well in predicting resting habitat and indicated that proximity to deep water foraging areas, depth, the proportion of bays with shallow depths, and rugosity were important predictors of spinner dolphin habitat. Predicted locations of suitable spinner dolphin resting habitat provided in this study indicate areas where future survey efforts should be focused and highlight potential areas of conflict with human activities. This study provides an example of a presence-only habitat model used to inform the management of a species for which patterns of habitat availability are poorly understood.

## Introduction

The study of species- environment relationships can provide important insight into the processes underlying a species' habitat use and distribution. Accurately describing species' distributions is critical to developing effective conservation efforts [1], [2], [3], [4]. In particular, species distribution models (SDMs) can provide quantitative predictions of geographic distributions and are increasingly being used to address a wide range of ecological questions [4], [5], [6], [7]. SDMs are useful to conservation as they can be used to predict locations where species are likely to occur in areas that have not been surveyed or have been poorly surveyed. This allows: 1. future surveys to be focused in areas where species are likely to occur; 2. species data to be evaluated relative to habitat alterations; and 3. high-priority sites for conservation to be identified [8].

Traditional SDMs have relied on presence/absence data from standardized surveys [9], [10], [11]. When survey effort data are available, pseudo-absences generated from surveyed areas can be used along with occurrence data in presence-absence models such as generalized linear models (GLMs), generalized additive models (GAMs), or Classification and Regression Trees (CARTs) [4]. However, the use of pseudo-absences presents limitations; while species presences can be confirmed, species absences can be difficult to document with certainty, particularly for mobile species, and increased sampling effort must be performed in order to ensure the reliability of absence data [12]. The efficiency of different survey methods can vary, and can also lead to sampling error due to non-detection [13]. False absences included within presence-absence predictive models can decrease the reliability of these models [13]. Significantly, available data for many species of conservation concern have been collected opportunistically and/or from a variety of platforms, and datasets derived from systematic surveys are often limited or incomplete.

Presence-only records from sources such as museum collections, herbariums, or online databases are becoming increasingly available and provide valuable resources for modeling efforts [1], [14]. Developments in modeling techniques have allowed these data to be used to predict species' distributions despite a lack of confirmed absences in areas that were surveyed in which no species observations were made. Maximum Entropy modeling, or Maxent, is a presence-only modeling technique that has recently been applied to ecological studies [15], [16], and has been found to perform well in comparison to established modeling techniques [6], [8]. Maxent offers several advantages over conventional modeling techniques evaluating the habitat use of a particular species. As a presence-only technique, Maxent allows species distributions to be modeled when no data on species absences are available. Presence-only techniques such as Maxent are particularly useful for studies of species with large ranges and low sightings, as for many cetacean species, for regions where systematic surveys are sparse and/or limited in coverage, and for datasets for which absence or effort data are not available [14], [17]. Since available sightings data often come from a variety of sources and survey platforms, as in the present study, Maxent can be used to provide robust models using opportunistic data from multiple platforms [17]. In addition, Maxent provides a flexible modeling approach in which both categorical and continuous variables can be applied, and provides a continuous output of predicted species distributions, allowing habitat suitability to be visualized and contrasted at a fine scale across a study area. Lastly, this method allows the relationship between predictor variables and model gain to be assessed graphically and quantitatively. Determining which habitat variables are the most important and illustrating how they affect species distributions in a mapping environment is particularly useful for managers charged with ensuring their sustainability.

Available data describing spinner dolphin (*Stenella longirostris*) habitat in the main Hawaiian Islands provide a good example of the utility of Maxent. Identifying and quantifying spinner dolphin habitat within bays in the main Hawaiian Islands is critical to determining how the effects of tourism and other human activities might impact wild spinner dolphin populations in the future. While standardized cetacean surveys have been conducted in some bays of the main Hawaiian Islands, particularly on the island of Hawai'i (e.g., Wailea Bay, Kealakekua Bay), many bays have received little survey coverage, or have only been surveyed opportunistically during tagging or focal follow studies for which data have not been published. A comprehensive survey of all bays in the main Hawaiian Islands would be logistically demanding and expensive, and predictive models are a cost effective alternative for directing surveys in order to identify unknown habitat and to quantify available habitat. Locations of predicted habitat can then be related to areas of increased human activity and can be used to indicate regions where conservation measures should be focused.

Spinner dolphins are relatively small dolphins (ca. <250 cm, [18]) named for their aerial behavior and are found in subtropical and tropical oceans around the world. There is wide variation in the morphology and color patterns of spinner dolphins throughout their range, and four subspecies of spinner dolphins are currently recognized: *S.l. longirostris* (Gray's spinner), *S.l. orientalis* (Eastern spinner), *S.l. centroamericana* (Central American spinner) and *S.l. roseiventris* (Dwarf spinner) [19], [20]. The Gray's spinner dolphin is the most widely distributed sub-species, and occurs throughout the Hawaiian Archipelago. Recent genetic analyses have demonstrated that spinner dolphins in Hawai'i are significantly distinct from spinner dolphins found in other parts of the world, and genetic distinctions exist between subpopulations within the Hawaiian Archipelago [21], [22].

Spinner dolphins in Hawai'i show predictable daily movement patterns, tracking vertical and horizontal migrations of prey organisms in the mesopelagic boundary layer during nighttime hours (primarily myctophid fishes, small crustaceans and squid) [23], [24] and then moving into protected inshore areas to rest during daylight hours. This diel behavioral pattern appears common to spinner dolphins throughout tropical Pacific Islands (e.g., American Sāmoa, Moorea) [25], [26], occurs in other oceans [27] and has been most extensively studied in the Hawaiian Archipelago [28], [29], [30], [31]. Within the main Hawaiian Islands, the habitat use of spinner dolphin resting bays has been best documented on the west coast of the island of Hawai'i, and similar patterns of habitat use have been documented along O'ahu and in the Northwestern Hawaiian Islands [29], [30], [31]. Spinner dolphins typically enter protected bays of the main Hawaiian Islands just after dawn, and slowly descend into a resting state over a period of up to two hours. The resting state is defined by slow movements, a cessation of aerial behavior, synchronous dives by tight groups of dolphins that are touching or almost touching, and visual, rather than acoustic, vigilance [29]. Norris and Dohl (1980) suggested that the formation of these tight, synchronized groups of resting dolphins might enhance their ability to detect and react to predators while the animals are not actively echolocating [28]. Groups of resting dolphins typically move slowly within bays for four to five hours, after which dolphins undergo a period of “zig-zag swimming” and increase surface activity before moving into deeper waters near sunset to begin night-time foraging. This behavior is thought to be a form of social facilitation ensuring alertness and group synchrony for foraging bouts.

The behavioral patterns of spinner dolphins in Hawai'i and their dependence on shallow coastal habitat during the day may make this species particularly vulnerable to impacts of human activities. The growth of lucrative dolphin-based tourism in the main Hawaiian Islands [32] and the increase in human/dolphin interactions in recent years [33] has only reinforced the original concerns of Norris et al. (1994) regarding the overlap of these activities with the resting habitat of spinner dolphins. Increases in human/dolphin interactions have resulted in negative impacts on dolphin populations in other parts of the world [34], highlighting the potential effects of disturbance due to tourism on the habitat use of cetaceans. Disturbing resting spinner dolphins may greatly affect their distribution and behavior [31] and may have caused population-level effects on these animals that remain undetected in the absence of long-term studies.

Although the daily patterns of spinner dolphin movements have been documented in detail [28], [29], the factors influencing how spinner dolphins choose resting habitats remain unclear. Spinner dolphins appear to use only certain bays as resting habitat, and are thought to select shallow, calm, flat, protected, sandy bays that provide easy access to deep water foraging areas [29]. Within these bays, spinners are thought to prefer areas with depths of less than 50 m. Bay area is also believed to be an important factor affecting spinner dolphin resting habitat, as bays with larger areas of suitable habitat may have a larger “carrying capacity” for resting spinner dolphins [29]. However, these original hypotheses have never been tested quantitatively and many bays within the main Hawaiian Islands have not been comprehensively surveyed for the presence of resting spinner dolphins. As a result, the distribution of spinner dolphin resting habitat and the relative importance of different resting bays are not well understood.

The goal of this study was to use available data to quantitatively test the previously hypothesized environmental factors that contribute to spinner dolphin resting habitats and predict the locations of resting habitat in the main Hawaiian Islands. The output of this habitat model will be useful in informing management regarding the current overlap of human activities and potential spinner dolphin resting habitat. In addition, the results of this study can be used to evaluate the potential for future conflict between spinner dolphin resting habitat and human activities with the continued increase of tourism and other human activities in the Hawaiian Islands.

## Materials and Methods

### Study Area and Time Frame

**Figure 1:**
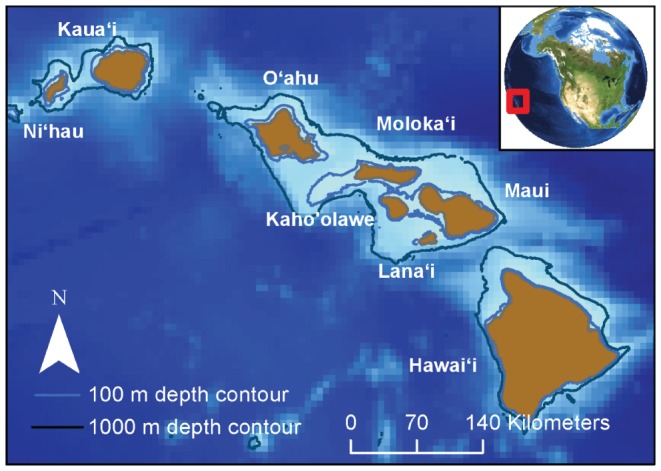
Location of the study site in the Hawaiian Archipelago.

The analysis of spinner dolphin resting habitat was restricted to bays of the main Hawaiian Islands following previous observations of spinner dolphin resting behavior in these areas [28], [29]. Eight islands comprise the main Hawaiian Islands, which range in size from approximately 130 to more than 10300 km^2^ and span a distance of approximately 650 km (Figure 1). Data used in this analysis were collected between 2000 and 2010. Sightings recorded within the same day were defined using a modification of the “chain rule” [35], whereby a dolphin within 100 meters of any other member of a group of dolphins was considered to be a member of that group.

### Data

Bays in the main Hawaiian Islands were digitized manually in ArcGIS version 10.0 by selecting indentations in the coastline greater than 2000 m in length (Figure 1). When less than approximately 75% of a bay contained environmental data (described below), the bay was excluded from the analysis. A total of 99 bays were included in the analysis; 46 bays were excluded due to insufficient environmental data. Environmental variables were selected to reflect factors previously hypothesized to be important to resting spinner dolphins [28], [29] and based on the availability of continuous data throughout the main Hawaiian Islands.

Presence-absence data for spinner dolphin surveys were not available. Locations of spinner dolphin sightings were obtained from the Pacific Islands Photo-Identification Network (PIPIN) catalogue and a variety of other data archives, and included sightings from aerial, boat-based and land-based surveys (Table 1). Although further sightings data are known to exist [29], the exact sighting locations (x, y coordinates) of dolphins from these studies were not available. Sightings that were located within the digitized bays and had the following behavioral states were assumed to represent resting spinner dolphins and were included in the analysis: rest, mill, slow travel, or not surface active. Sightings that did not include a behavioral description or that represented active dolphins (e.g., travelling, leaping/spinning, bow riding) were excluded from the analysis. Although some animals within a group of resting spinner dolphins have been observed to bow ride, if the behavioral state of the group of animals was characterized as “bow riding”, the sighting was excluded from the analysis. This restricted our analysis to 225 of the 497 spinner dolphin sightings available in the database. Although spinner dolphin sightings in the database included sightings throughout the main Hawaiian Islands, most sightings used in the model (174 of 225) were collected from bays on the island of Hawai'i, which is considerably larger than the other islands (Figure 1) and where available resting habitat is thought to be particularly prevalent [28], [29].

**Table 1:** Number of spinner dolphin sightings by survey platform. See text regarding the selection of sightings used in the model.

Spinner dolphin sightings	Aerial	Boat-based	Shore-based	Total sightings
Total sightings in database	14	452	31	**497**
Sightings used in model	3	193	29	**225**

We used three variables to assess the benthic relief within bays, both to provide a proxy for bottom type [36] and to investigate hypotheses of dolphin preference for bottom habitat more broadly. We used continuous surfaces of topographic slope to evaluate bathymetric gradients within bays, along with rugosity, a measure of the roughness of the bottom [37], and aspect variety, a measure of the heterogeneity in the downslope directions, to assess benthic relief. Rasters of bathymetric slope and downslope direction were generated from 50 m bathymetric grids obtained from the Hawai'i Mapping Research Group (School of Ocean and Earth Science Technology, University of Hawai'i at Mānoa; http://www.soest.hawaii.edu) using the Spatial Analyst extension in ArcGIS 10.0. Aspect variety assessed variety in downslope directions within a 5×5 cell neighborhood. Rugosity was defined as the ratio of the surface area to the planimetric area [37], calculated using the ArcGIS extension, Surface Areas and Ratios from Elevation Grid v. 1.2 (). The spatial scale of these measures of benthic relief was similar to that used by Dunn and Halpin (2009) to model bottom habitat using indices generated from depth coverages [36].

**Figure 2:**
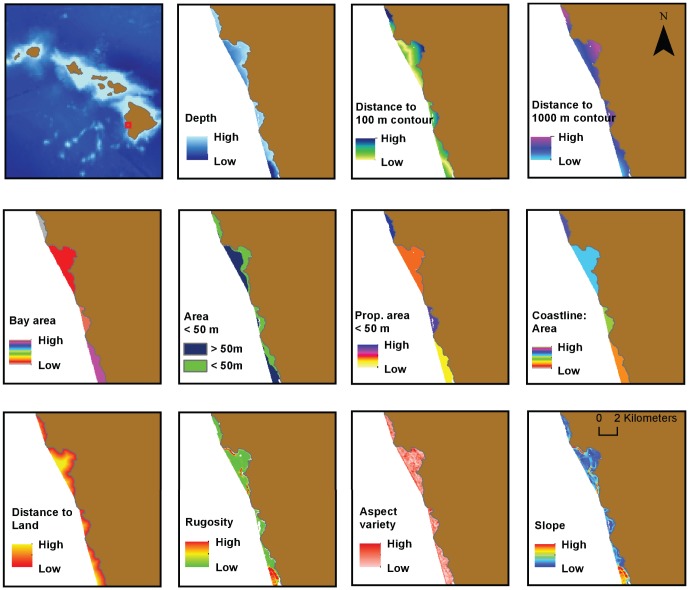
Examples of environmental variables used to model spinner dolphin resting habitat within bays of the main Hawaiian Islands.

Both fine-scale variables, calculated at the location of spinner dolphin sightings, and bay-level variables, such as bay area, were included in the analysis. Multiple spinner dolphin sightings were located within a single bay and thus bay variables were categorized into even classes to avoid spurious species-variable relationships due to identical values for multiple sightings. For example, the proportion of bay area under 50 m was divided into the following five categories: 0 to 0.19; 0.20 to 0.39; 0.40 to 0.59; 0.60 to 0.79; and 0.80 to 1. The following variables were calculated at the location of each spinner dolphin sighting: depth; distance to 100 m and 1000 m depth contours; distance to land; rugosity; slope; and aspect variety. Bay variables for each sighting included bay area; the ratio of coastline to area of a bay; the total bay area at depths of less than 50 m; and the proportion of area with depths of less than 50 m. The distance to the 1000 m contour was used to represent distance to deep-water foraging habitat. In some locations spinner dolphins have been observed to forage in waters with a depth of <100 m near midnight when their prey reach the peak of their vertical migration [23]. However, spinner dolphins appear to follow their prey into deeper waters during nighttime hours before and after midnight [23], and the 100 m contour was selected to represent the inshore region of spinner dolphin foraging habitat. The ratio of coastline to area of each bay was used as a proxy for protection; bays with high coastline to area ratios were more concave and thus were presumed to be more sheltered from offshore wind and waves. Relationships between environmental variables were evaluated using Pearson's correlation coefficients to identify correlated variables that could not be analyzed within the same model. All map layers were projected using a Mercator projection prior to analysis. Figure 2 shows an example of each map layer that was generated for the analysis.

### Maximum Entropy modeling

Maximum Entropy modeling was performed to provide probabilistic predictions of spinner dolphin resting habitat. The Maximum Entropy technique has its roots in information theory [38] and has been used as a statistical modeling method in several fields, particularly in natural language processing [39]. Recently, Maximum Entropy modeling has been applied to predictive modeling of species distributions [6], [15], [16], [40], including small cetaceans [41]. We used the Maxent program (version 3.3.1 – see http://www.cs.princeton.edu/~schapire/maxent) as described in detail in Phillips et al. (2006, 2009) [16], [41]. Briefly, Maxent employs a maximum likelihood method that models species' distributions by generating a probability distribution over the pixels in a grid of the study area. Maxent estimates a probability distribution that maximizes entropy (i.e., that is the closest to uniform) subject to a set of constraints derived from measurements of assumed suitable habitat values at species occurrence locations. Specifically, the expected value of each environmental variable of the Maxent distribution must match its empirical mean (the mean over the sample points). The probability distribution is estimated over the pixels of the study area, and the pixels representing species presences make up the sample points. During a model run, the “gain” represents the probability distribution of the model and is a measure of the likelihood of the samples. The gain starts at 0 and increases with every model iteration until the difference between model iterations is below the convergence threshold. The gain can be thought of as a measure of how much better the distribution fits the sample points in comparison to the uniform distribution, and is similar to the “deviance” used in statistics. Maxent uses regularization techniques to smooth resulting models to ensure that models are not overfit [6], [14], [16], and we used a constant regularization parameter set to the default value of 1 (higher regularization values would produce smoother models, which we did not require) [7], [14].

Phillips et al. (2009) note that occurrence data is often spatially biased towards particular areas, such as those that are easily accessible, while background data used to build presence-only models are typically based upon randomly drawn data [41]. This difference in the spatial bias between occurrence data and background data can cause resulting models to be inaccurate. This problem can be overcome by using background data with a similar bias as the occurrence data (the use of target-group background data). Phillips et al. (2009) found that this approach improved model performance considerably [41]. We used target-group background data to build our model of spinner dolphin resting habitat. We examined the location of all of the available presence-only data (both resting and non-resting spinner dolphins, as well as sightings within and outside of the bays) and identified bays that were therefore considered to have been surveyed (all bays that either contained sightings, regardless of the behavioral state of the dolphins, or bays that were within 1000 m of a sighting). We used background data from within only these bays to build the model, and then applied the model to all bays within the main Hawaiian Islands.

We used cross-validation to assess model fit. To cross-validate the model, spinner dolphin sightings were randomly split into groups of equal size and multiple models were created (10 replications in total), leaving out each group in turn. This allowed variance estimates to be produced from the different Maxent model runs and evaluated relative to the average results across all models [14], [42], [43], [44]. Cross-validation is advantageous for small datasets as it uses all of the data, rather than splitting the data into test and training groups, and has been found to be a preferable method of model assessment [45].

Maxent provides both threshold-dependent and threshold-independent measures of model outputs. Threshold-independent assessments are evaluated using the Area Under the Curve (AUC) metric of the Receiving Operator Characteristic (ROC) curve [46]. In an ROC curve, all sensitivity values (true positives) are plotted on the *y*-axis against specificity (false positive) values on the *x*-axis. The AUC value provides a threshold-independent metric of overall accuracy, and ranges between 0.5 and 1.0. Values of 0.5 indicate that scores of specificity and sensitivity do not differ, while scores of 1.0 indicate that the distributions of the scores do not overlap [46]. We evaluated AUC values of the ROC curve of the model as in Hosmer and Lemeshow (1989): <0.5 indicated no discrimination; 0.5 to 0.7 represented poor discrimination; 0.7 to 0.8 indicated an acceptable discrimination; 0.8 to 0.9 indicated an excellent discrimination; and >0.9 represented outstanding discrimination [47]. We also evaluated whether the model predicted spinner dolphin sightings significantly better than a random prediction with the same fractional predicted area using one-tailed binomial tests (threshold-dependent assessments). Maxent output is typically provided as a probability of species occurrence, and a threshold value must be provided in order to generate presence-absence results. The equal training sensitivity and specificity logistic threshold, which has been found to perform better than other commonly used thresholds [48], was applied and compared with the results from fixed thresholds of 1, 5, and 10. The performance of the model at these thresholds was then assessed using the extrinsic omission rate and the proportional predicted area. The extrinsic omission rate is the fraction of spinner dolphin sightings that occur on pixels that are not predicted to be suitable for the species, while the proportional predicted area is the fraction of pixels that are predicted to be suitable habitat [16]. Lastly, we examined variable importance within Maxent models using a jackknife analysis. Models were computed repeatedly leaving out one variable at a time, and then creating a model using each variable in isolation. This allows the contribution of each variable to the model to be computed individually, and also allows the model performance to be assessed when each variable is not included in the analysis.

Species distributions were modeled using Maxent version 3.3.1. When habitat variables were highly correlated (significant Pearson's correlations greater than 0.50), only one of the correlated variables was included in the final Maxent model based on the potential biological relevance of the variables [49]. Pearson's correlation coefficients for model variables are shown in Table 2. The final model included the following variables: aspect variety, bay area, coastline to area ratio, depth, distance to the 100 m contour, proportion of bay area with depths <50 m, and rugosity.

**Table 2:** Pearson's correlation coefficients for model variables. Coefficients shown in bold represent significant correlations greater than 0.5.

	Depth	Area	Bay area <50 m	Prop. area <50 m	Dist. 100 m cont.	Dist. 1000 m cont	Dist. land	Slope	Rug.	Asp. Var.	Coast: area
**Depth**	–	0.24	0.01	0.46	0.35	0.24	−0.45	**−0.68**	−0.47	0.17	0.24
**Bay area**		–	**0.78**	−0.24	−0.12	0.14	**0.68**	−0.08	−0.06	−0.04	−0.31
**Bay area <50 m**				0.07	−0.09	0.07	**0.61**	−0.20	−0.12	0.05	−0.50
**Prop. area <50 m**				–	0.38	0.04	−0.22	−0.49	−0.36	0.14	0.35
**Dist. 100 m cont.**					–	**0.59**	−0.22	−0.36	−0.23	0.00	−0.12
**Dist. 1000 m cont.**						–	0.01	−0.31	−0.19	0.04	0.14
**Dist. land**							–	0.04	0.02	−0.10	**0.64**
**Slope**								–	**0.62**	−0.17	−0.03
**Rug.**									–	−0.11	−0.10
**Asp. Var.**										–	−0.04
**Coast: area**											–

## Results

**Figure 3:**
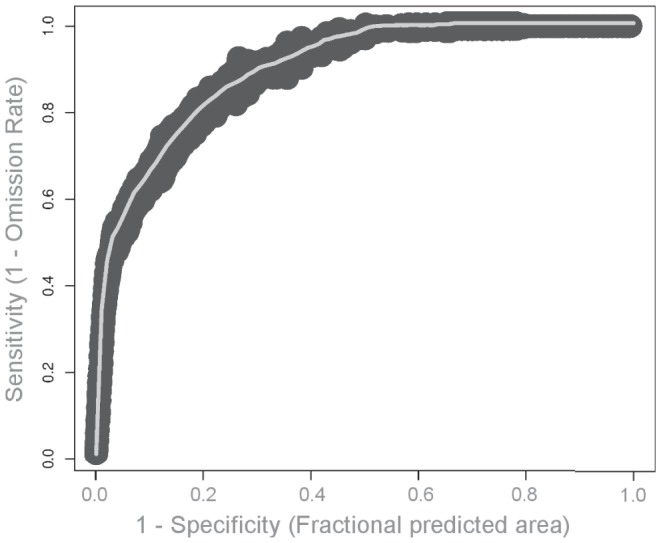
Receiver Operating Characteristic (ROC) curve and the Area Under the Curve (AUC) values for training and test data.

For the threshold dependent tests, p-values of binomial tests for all thresholds evaluated were ≫0.01, indicating that the model predicted test localities significantly better than random (Table 3). When binary output is desired (e.g., habitat vs. non-habitat), the threshold value used becomes critical, and further research is required to establish rules for choosing optimal thresholds to distinguish suitable habitat from unsuitable habitat [7], [16]. Threshold independent tests of the model also indicated that the model performed well in predicting spinner dolphin resting habitat. The mean AUC value for the cross-validated model was 0.87, which was considered to offer “excellent discrimination” given our interpretation of AUC values (see Methods section; Figure 3).

**Table 3:** Fractional predicted area and *p*-values of binomial tests from the Maxent model of spinner dolphin resting habitat for the equal sensitivity-specificity threshold and for fixed thresholds of 1, 5 and 10.

Description	Fractional predicted area	*P*-value
Fixed cumulative value 1	0.578	2.39×10^−9^
Fixed cumulative value 5	0.382	6.79×10^−12^
Fixed cumulative value 10	0.292	2.27×10^−14^
Equal training sensitivity and specificity	0.189	7.34×10^−16^

**Figure 4:**
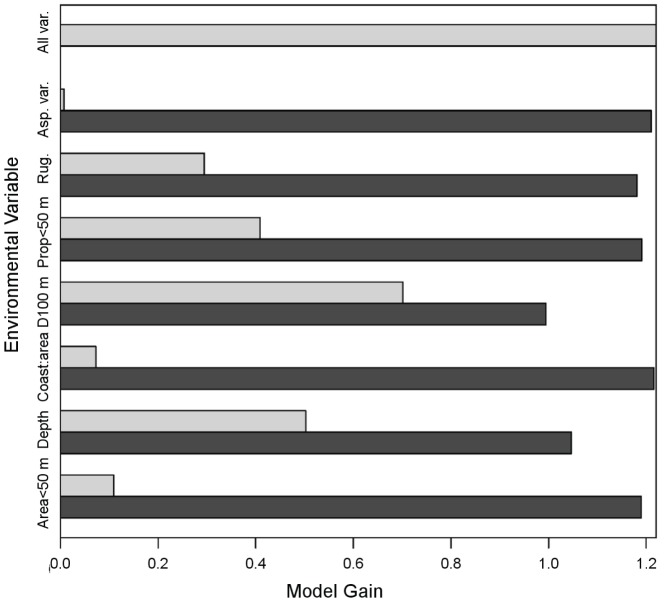
Results of Maxent model showing jackknife tests of variable importance for training samples.

Results of a jackknife test of variable importance in the final model run are shown in Figure 4. Of the variables, distance to the 100 m depth contour, depth, the proportion of bay area with depths of less than 50 m and rugosity were found to be the strongest predictor variables. Total bay area with depths of less than 50 m, coastline to area ratio and aspect variety were relatively weak predictors.

**Figure 5:**
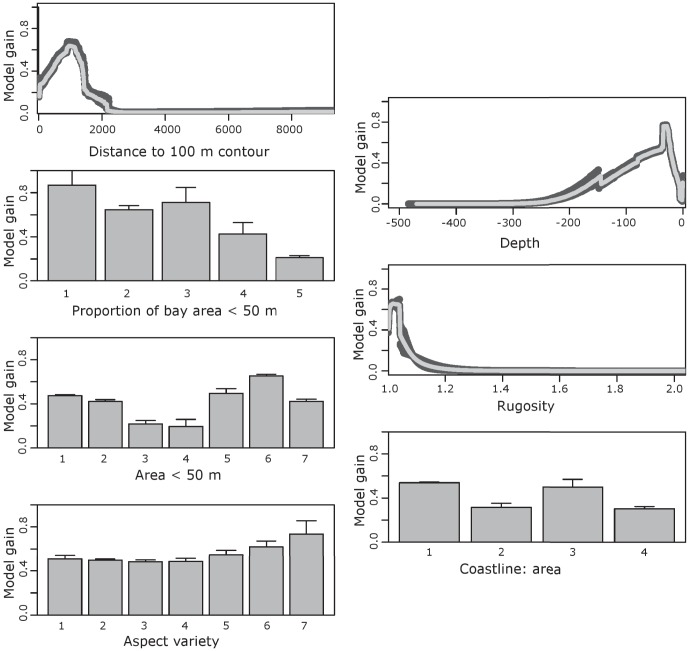
Response curves (+/−1 standard deviation) showing how each of the environmental variables included in the model affects the Maxent prediction.

Maxent model responses to the different environmental variables are shown in Figure 5. Distance to the 100 m depth contour showed a negative response, with the highest values of model gain occurring at distances of less than approximately 1.5 km. Depth also showed a negative response, with the highest model gain occurring between depths of approximately 15 to 50 m. Bays with a low proportion of area covered by depths of less than 50 m showed the highest values of model gain. The lowest values of rugosity showed the highest values of model gain. Both low and high categories of bay area under 50 m showed a positive response. Low or medium values of coastline to area ratio and higher values of aspect variety were associated with increased model gain.

**Figure 6:**
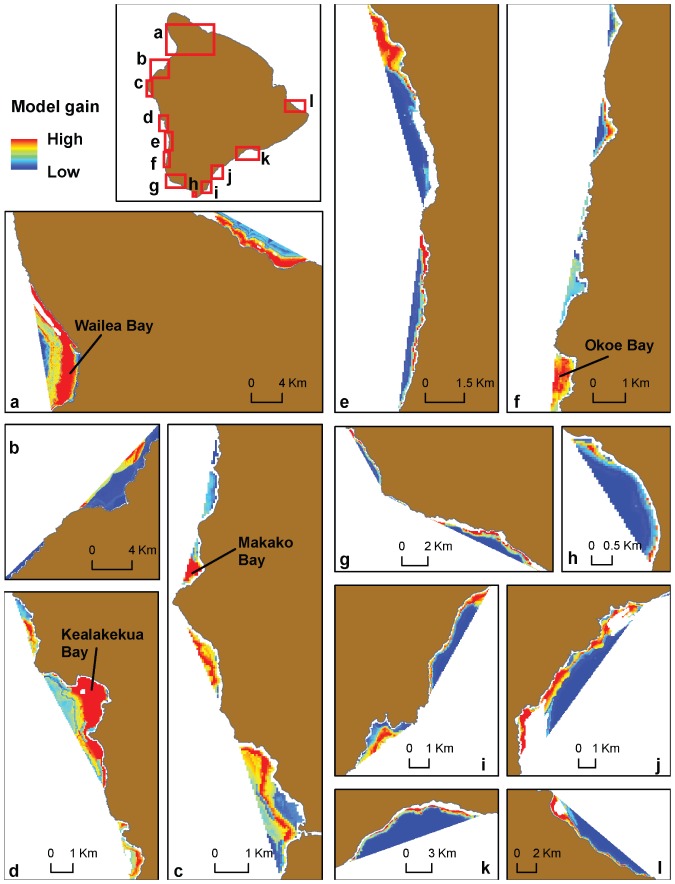
Model gain shown for selected bays on the island of Hawai'i.

**Figure 7:**
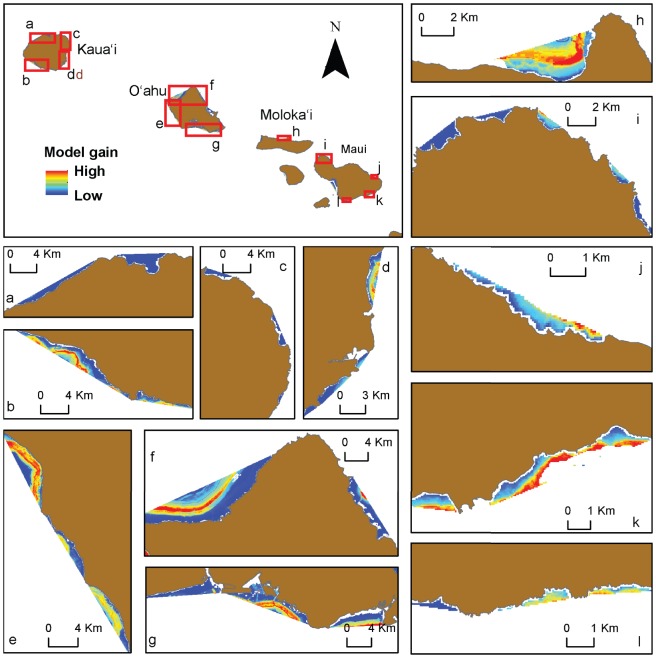
Model gain shown for selected bays on the islands of Kaua'i, O'ahu, Moloka'i and Maui.

**Figure 8:**
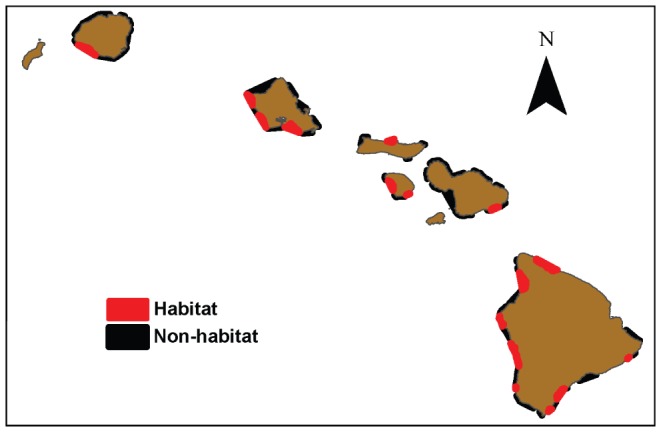
Example of spinner dolphin resting bays predicted from model output identified using the maximum sensitivity plus specificity threshold (see text).

**Figure 9:**
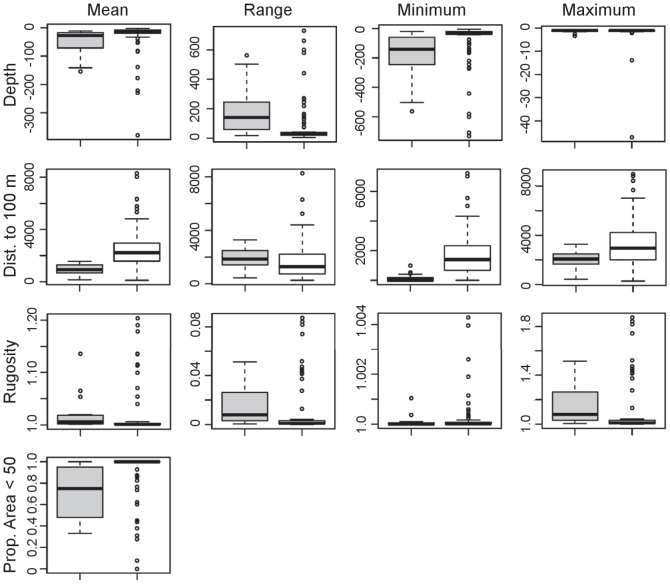
Boxplots of strongest predictor variables for spinner dolphin habitat in bays identified as habitat (shown in grey) and non-habitat (shown in white) using the equal sensitivity-specificity threshold.

The mean spatial model of the predicted resting habitat for spinner dolphins is shown for selected bays on each island in Figures 6 and 7. Although we emphasize that results from models using thresholds for binary output should be interpreted with caution, we also provide an example of how our model results can be evaluated in terms of habitat vs. non-habitat to simplify the model for demonstration purposes. We used the most conservative threshold value produced from the model (i.e., that giving the lowest predicted area), which was the threshold producing equal values of sensitivity and specificity (Table 3), to identify spinner dolphin resting habitat. Bays that were found to contain a considerable amount of predicted habitat using this method (here defined as more than 25% of the total bay area) are shown in Figure 8. Using this method, 21 of the 99 bays evaluated were identified as potential spinner dolphin resting habitat. Potential resting bays were particularly prevalent on the western coast of the island of Hawai'i and on the southern and southwestern coasts of O'ahu. Boxplots of the most important model variables (depth, distance to 100 m contour, proportion of area <50 m, and rugosity) were produced at the bay scale (i.e., averages over bays) to compare values of these variables between bays identified as habitat and non-habitat using the equal sensitivity-specificity threshold (Figure 9). These boxplots examining spinner dolphin habitat at this larger spatial scale illustrated that bays considered to be spinner dolphin habitat showed deeper depths, lower distances to the 100 m contour, slightly higher values of rugosity, and lower proportions of area under 50 m in comparison to bays not classified as spinner dolphin habitat.

**Figure 10:**
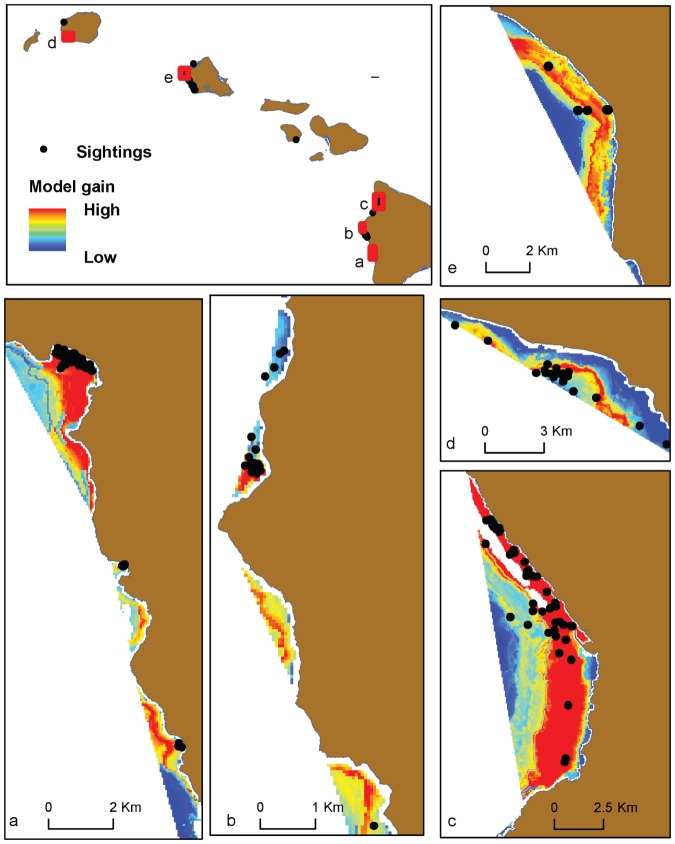
Examples of spinner dolphin sightings used to generate the model relative to model gain (probability of predicted spinner dolphin resting habitat).

An examination of the model output with the spinner dolphin sightings used to build the model showed a good fit with the location of the sightings (Figure 10). The model did not appear to be overfit to the sightings (i.e., the prediction was not closely fit to the presence records with a very localized prediction), and predicted a high probability of spinner dolphin resting habitat in several areas where sightings were not available.

## Discussion

### Environmental predictors of spinner dolphin resting habitat

The Maxent model performed well in predicting spinner dolphin resting habitat. Our results further confirm that Maximum Entropy modeling is a useful technique for predicting species distributions in situations where only presence data are available and where management of the species in question would benefit from a quantitative habitat analysis. Our model results indicated that proximity to deep water foraging areas, depth, the proportion of bays with shallow depths, and rugosity were important predictors of spinner dolphin habitat. Proximity to nighttime foraging areas has been proposed as an important factor affecting the use of bays by resting spinner dolphins [29]. The results of the present study confirm this hypothesis quantitatively; the jackknife test of variable importance indicated that the strongest predictors of spinner dolphin resting habitat were distance to the 100 m depth contour and depth, with spinner dolphin resting habitat generally occurring in shallow depths that were close to the 100 m depth contour. The importance of the distance to 100 m contour variable indicated that proximity to deep water was an important factor in predicting spinner dolphin habitat. Spinner dolphins in Hawai'i are primarily nighttime foragers and feed on the mesopelagic boundary community [23], [24]. The mesopelagic boundary community in this region has been found to consist of a distinct island-associated community of mesopelagic fish, shrimp and squid that occur along a narrow band at the boundary between the mesopelagic environment and the island slopes [50]. Recent studies have shown that in addition to a diel vertical migration in prey items (rising at night and returning to deep waters during daylight hours), a diel horizontal migration in the mesopelagic boundary layer also occurs in the main Hawaiian Islands. The mesopelagic boundary community migrates from deep, offshore waters into shallower, inshore waters at night and spinner dolphins appear to follow the diel horizontal migration of their prey [23]. The maximum foraging depth of a spinner dolphin is thought to be approximately 200–250 m [51] so the boundary community, located at depths of approximately 400–700 m during daylight hours [50], cannot be exploited by spinner dolphins during the day. Thus, using coastal resting areas proximate to deep waters would allow spinner dolphins to access to mesopelagic prey at an earlier stage in the diel migration of prey species into shallow waters. This would decrease energetic costs associated with traveling to deep waters where prey first become accessible to spinner dolphins, and would provide spinner dolphins with access to prey for a larger proportion of the night. Thus, access to nighttime foraging areas provides an ecological context to explain why animals might choose particular bays.

Previous studies [28], [29] hypothesized that spinner dolphins select flat bays with shallow depths and prefer shallow areas within these bays. Our model results examining bathymetry and rugosity support these original hypotheses, and move towards a mechanistic definition of spinner dolphin resting habitat during the time that these data were collected. Shallow depths were associated with resting habitat, though bays with a low proportion of area with depths less than 50 m were correlated with spinner dolphin resting habitat. Our model results suggest that spinner dolphins may select shallow areas within bays that encompass deeper waters (i.e., with a low proportion of area with shallow depths) so as to avoid predators while still maintaining proximity to offshore foraging areas. Rugosity was found to be a good predictor of spinner dolphin habitat. Low values of rugosity, indicating a low bottom roughness, were associated with spinner dolphin habitat. Heithaus and Dill (2002) suggest that dolphin echolocation is less efficient in shallow waters where sound is easily scattered off the bottom substrate and the surface of the water, contributing to a decreased ability to detect predators and an increase in the riskiness of such habitats for dolphins [52]. Similarly, we suggest that spinner dolphin echolocation might be less efficient in regions of high bottom roughness, causing dolphins to avoid more “risky” regions of high rugosity. Cluttered environments have been shown to impose considerable ecological constraints for echolocating bats [53], and high bottom roughness might create a cluttered acoustic background, affecting dolphins' ability to detect, classify, and locate predators and causing spinner dolphins to seek out regions of low bottom roughness. Furthermore, Heithaus and Dill (2002) suggested that tiger sharks are better camouflaged when swimming over seagrass habitats than when swimming over light sandy bottoms. The lower rugosity values in spinner resting habitat modeled in this study may reflect a similar relationship, where spinner dolphins are choosing flat (and likely sandy, as discussed below) resting areas within bays that increase their ability to visually detect shark predators while reducing acoustic clutter.

Multivariate models of rugosity evaluated at a similar scale to that used in the present study have been found to correlate well with estimates of bottom type [36], with high values of rugosity being associated with hard bottom substrates. Therefore our results showing that low rugosity was a good predictor of dolphin habitat support Norris and Dohl's (1980) hypothesis that spinner dolphins prefer bays with sandy bottoms [28]. Available high resolution bottom type data (http://ccma.nos.noaa.gov/) would have been useful in addressing hypotheses regarding spinner dolphin preference of bottom types but was often restricted to only the innermost regions of the bays. Due to this inconsistent coverage, these data were not used in the analysis. The other proxy for benthic complexity used in the present study, aspect variety, was not found to be an important predictor of spinner dolphin resting habitat. The effect of scale on proxies for benthic complexity should be examined further in the main Hawaiian Islands. These factors might be more appropriate to the current application if assessed with finer-scale data. Light Detection And Ranging (LIDAR) radar data provide high-density bathymetric data that have been used to provide estimates of rugosity across a range of spatial scales [54] and can be useful in studies of animal-habitat relationships [55]. LIDAR data would provide higher resolution bathymetric data than that used in the present study but are restricted to the innermost reaches of spinner dolphin resting bays. Field measurements of bottom type and fine-scale bathymetry should be included in future studies in order to address hypotheses regarding the importance of benthic topography and bottom type on spinner dolphin resting habitat.

Boxplots comparing bays classified as spinner dolphin habitat using the equal sensitivity-specificity threshold to bays classified as non-habitat demonstrated marked differences in physical characteristics between these bays. Bays classified as spinner dolphin habitat were closer to the 100 m contour than non-habitat bays, and showed a low proportion of area with depths of less than 50 m, which is consistent with the proposed importance of distance to deepwater foraging areas as discussed above. Deeper depths and slightly higher values of rugosity were observed in bays containing spinner dolphin habitat, though model results indicated that spinner dolphin habitat is associated with shallow depth and low rugosity. Since boxplots were produced using data at the bay scale (i.e., averaged over entire bays), this suggests that spinner dolphins are seeking regions with shallow depths and low rugosity within bays that include areas of deeper depths and higher rugosity than bays that do not contain spinner dolphin habitat. These bays are likely preferred due to their proximity to deepwater areas.

### Spatial predictions of spinner dolphin resting habitat

A visual examination of the model output matched well with known locations of spinner dolphin resting habitat that were not represented in the species occurrence data used for this model. For example, exact locations of spinner dolphin sightings within known resting bays such as Okoe Bay on the island of Hawai'i were not used to build the model. However, our model predictions indicated that these bays have a high probability of resting habitat (e.g., Figure 6f), providing an additional qualitative test of the model. Additional surveys providing exact locations of sightings within these known resting bays would be useful in improving the current model. The current analysis focused on bays of the main Hawaiian Islands, though Lammers (2004) observed resting spinner dolphins offshore of the bays used in this analysis along the southern shore of O'ahu [31]. The model output from the present study also indicated that offshore regions of bays along the southern shore of O'ahu had a high probability of spinner dolphin resting habitat. Lammers (2004) also observed resting spinner dolphins within the bays along the western shore of O'ahu in regions that showed a high probability of being spinner dolphin resting habitat in the Maxent model [31].

### Applications for management

Spatial maps of model output showed that spinner dolphin resting was often predicted to occur in regions close to shore in popular tourist areas. For example, suitable spinner dolphin resting habitat was predicted immediately alongshore in several bays along the west coast of the island of Hawai'i, a very popular tourist destination where conflicts with human activities have already been reported [33]. There are few published studies on the effects of tourism on resting spinner dolphins, and most do not address the potential for population-level effects. Limited observations suggest that socially active spinner dolphins might be relatively tolerant of human presence [31], while resting spinner dolphins may leave an area if forced to interact with humans [29], [56]. Studies of the effect of spinner dolphin presence on the level of tourist activity on Hawai'i found that increased numbers of kayakers and swimmers were observed when spinner dolphins were present [57], highlighting the need to evaluate the impacts of tourism on resting spinner dolphins. Understanding the current habitat use of resting spinner dolphins is a necessary first step in evaluating and comprehending the effects of human activities on this species.

Repeated human disturbance might have impacts that are not evident, including reduced benefits of rest periods. Daily resting behavior may provide a period of relative silence, allowing for the maintenance of sound-producing structures [29]. The central nervous system is unable to remain attentive for long periods of time, and thus a vigilance decrement, in which animals gradually show a decreased ability to process information, is observed over long time periods [58]. This can result in a decreased performance in activities such as detecting predators and capturing prey. A vigilance decrement may be of particular concern for spinner dolphins, which are thought to be limited by foraging efficiency rather than prey availability [59]. Wild animals must maintain appropriate proportions of foraging and rest, and vigilance decrement may be a significant factor influencing the time budgets of wild animals [58]. In this context, the daily rest time of spinner dolphins likely represents an important period of vigilance recovery that is critical to their ability to function effectively in their oceanic foraging habitat. Human-driven shifts in habitat use to open water or less suitable habitats [14] might also have consequences for avoiding predation and vigilance decrement.

Maxent results are typically reported as probabilities rather than binary output (habitat vs. non-habitat), which has important implications for managers seeking to use SDMs to define or delineate regions of interest. We stress that the thresholds used to develop binary model output need to be evaluated carefully, but also suggest that this approach presents a replicable method for identifying important habitat that could be adapted depending on the management context or the perceived level of risk to a given species. In our example using the equal sensitivity-specificity threshold, only a small number of bays (21 of 99) were identified as providing suitable habitat for resting spinner dolphins, which highlights two points for the effective management of human activities in Hawai'i. Firstly, spatial modeling approaches such as the results presented here can be used to focus future survey effort in bays that have not been surveyed for spinner dolphins or for which no data is currently available, such as in bays on Lāna'i, Moloka'i, Maui or along the southeastern coast of the island of Hawai'i. Focusing survey efforts in this way would be useful in reducing costs of at-sea surveys and would provide a further test of our model in order to improve our understanding of the habitat required by resting spinner dolphins. Secondly, the finding that a low proportion of bays provide resting habitat for spinner dolphins suggests that detrimental effects of human activities on resting spinner dolphin habitat could be minimized by restrictions or preventative measures in a relatively small number of bays. The results of this study indicate the importance of using presence-only modeling techniques to evaluate the habitat use of species when limited data are available, or when no absence or effort data are available, particularly for species where model results can be used to address management concerns. Maxent models are especially informative in this respect as they perform well compared to other presence-only modeling techniques [6], [16], allow a variety of data types to be incorporated, present graphical relationships between predictions and environmental variables that can be easily understood by resource managers, and provide continuous spatial output of model predictions [16].

### Conclusions

In summary, this study further demonstrates the utility of Maximum Entropy modeling for mapping species distributions of species for which patterns of habitat availability are poorly understood. Results show a good fit with known areas of spinner dolphin resting habitat, and provide important information regarding the environmental factors affecting spinner dolphin habitat use. Maps of spinner dolphin resting habitat produced from this study can be used to focus further analyses of habitat use and to select areas where effects of human activities on resting spinner dolphins should be monitored in the future.
